# Mind a Murderer

**Published:** 1867-11

**Authors:** 


**MIND A MURDERER.**—It is not an unfrequent occurrence in the experience of eminent physicians to be consulted where there is no tangible evidence of actual disease. The man does not look like an invalid; on the contrary, he has the external, the physical appearance of good health. Yet he complains of a great variety of symptoms; and overshadowing them all is an exaggeration of actual sufferings with an oppressive foreboding of greater ones still to come. The appetite is good, but there is no elasticity of body and less of mind. The muscles are plump and of a healthful hue; or if lean and lank, there is no "lesion of parts," no inveterate disease of bone or blood. The general system, too, is in the regular performance of its functions; still the patient persists in the statement that he is "miserable."

There are similar cases in the religious world, in the experience especially of city clergymen. A man is a church-member. He stands well in his "society;" he is prosperous in business, and is considered an honorable, high-minded citizen; still he does not "enjoy religion," he is not a blithe-hearted man, gladsome and sunshiny. The experienced physician looks with apprehension on such a state of things, because he knows that the next step is to waste away, to wither and to die, and that these results will come on apace unless the causes of the mental malady are removed. To do this requires a fearlessness, a degree of moral courage which not many men possess. The state of things described arises from the condition of the mind, from mortification, from remorse, or despair. A gentleman of great wealth, in the full vigor of health and mental maturity, was charged with perjury, with a view of extorting a sum of money. The charge was made in a manner so peculiar, and with such plausibility, by reason of some coïncidences, wholly fortuitous, that there was no help for it but to go to jail and await a legal investigation. He was triumphantly acquitted, but the mortification was such that he sickened and died in a few days; all the organs of the body being found, on a post-mortem examination, to be in a most perfect and healthful condition.

All know that remorse can eat out a man's life in a short time; and so can despair of pardoned sin. The philosophy of such cases is, that great mental emotion of a depressing character greatly interferes with the depth of breathing. Every now and then nature makes a desperate effort for relief in the long-drawn sigh; but in the intervals, the person scarcely breathes at all, perceptibly; hence the blood is not supplied with its proper amount of air, which is the agent for relieving it of its impurities; hence also, the blood becomes thick, does not flow through the veins, becomes too abundant, distending the blood-vessels in every direction, oppressing and weighing down all the faculties — those of the brain in particular — and the hand is instinctively raised to the brow, as if to relieve it. A double illustration is found in the following well-authenticated fact. A man was sick; he was, in truth, slowly dying. He occupied a high position, but had no light, no joy. He deplored his sins and sought forgiveness; but there was no relief. His professional adviser became at length convinced that there was a malady of the mind which was at the foundation of all his trouble, and kindly but sternly said: "There is something undone, which you ought to do. God judge 'twixt you and it!" Fixing his eye intently on the speaker, the sick man arose in his bed and said: "Some years ago, I took passage for England. At the moment of sailing, a bag of money was handed to the captain for delivery; it was carelessly laid down, and rolled about on the locker from day to day. With the sole view of frightening him, I hid it. On reaching port, it was not missed. Months passed, and there was no inquiry for it. At length the captain was called on for the money. He remembered having received it, but could give no further account of it. Meanwhile I became alarmed, lest my character should be implicated, and deliberately hid the money. The captain was thrown into prison, where he languished for two years and died. By this time I became hardened — strove to stifle conscience; but the cares and strifes and amusements of the world were unavailing. Now I feel that there is no hope for me, and I must go down to the grave unpardoned." The troubled man was advised to hunt up the captain's widow, make restitution of principal and interest, and clear up the clouded reputation of her husband. This was promptly done. After restitution, accompanied by repentance, he passed quietly and even happily into the grave.

The lessons of this article are of great practical importance. First: it is a criminal weakness, because it endangers life, to allow the mind to be harassed by false reports and false charges. Go forward in doing right, knowing that God is your judge; that he is witness to your integrity, and that in due time he will, with increased honor, place you in your true position before all men. Second: if you have done your fellow-man a wrong; if you have unrightfully cast a stain on his character, even unwittingly; if you are withholding from him what is his due, against his consent, while you have ability to relieve yourself of the obligation, although the law may uphold you in the same, and conscience twinges you in these regards, do not waste time in criminal delays; do not deceitfully excuse yourself in the resolution to do even-handed justice at some future day, when you may be more able and would feel it less than now; let not another sun go down upon your criminality, and hope not for the "peace which passeth knowledge," until, with the truth and fervor and sincerity of a returning prodigal, of an humbled Magdalene, of the repentant thief, you make a "clean breast" of every thing, and thus cast the eating, the accursed leprosy from your heart forever; for an outraged conscience works death to the body as well as to the soul. Death was caused in the first case by mortified pride; in the second by remorse.

